# The Sound of Words Evokes Affective Brain Responses

**DOI:** 10.3390/brainsci8060094

**Published:** 2018-05-23

**Authors:** Arash Aryani, Chun-Ting Hsu, Arthur M. Jacobs

**Affiliations:** 1 Department of Experimental and Neurocognitive Psychology, Freie Universität Berlin, Habelschwerdter Allee 45, D–14195 Berlin, Germany; ajacobs@zedat.fu-berlin.de; 2 Department of Psychology, Pennsylvania State University, PA 16802, USA; hsuchunting@gmail.com; 3 Centre for Cognitive Neuroscience Berlin (CCNB), Freie Universität Berlin, Habelschwerdter Allee 45, D–14195 Berlin, Germany

**Keywords:** affective sound, affective potential of sound, sound-meaning, phonoaesthetics, sublexical arousal, unifying neural network, neurocognitive poetics

## Abstract

The long history of poetry and the arts, as well as recent empirical results suggest that the way a word sounds (e.g., soft vs. harsh) can convey affective information related to emotional responses (e.g., pleasantness vs. harshness). However, the neural correlates of the affective potential of the sound of words remain unknown. In an fMRI study involving passive listening, we focused on the affective dimension of arousal and presented words organized in two discrete groups of sublexical (i.e., sound) arousal (high vs. low), while controlling for lexical (i.e., semantic) arousal. Words sounding high arousing, compared to their low arousing counterparts, resulted in an enhanced BOLD signal in bilateral posterior insula, the right auditory and premotor cortex, and the right supramarginal gyrus. This finding provides first evidence on the neural correlates of affectivity in the sound of words. Given the similarity of this neural network to that of nonverbal emotional expressions and affective prosody, our results support a unifying view that suggests a core neural network underlying any type of affective sound processing.

## 1. Introduction

When communicating, humans usually express emotion through two different signaling systems: verbal vocalization, i.e., relating the semantic content of particular phoneme combinations (words), and nonverbal vocalization, i.e., relating paralinguistic cues such as intonation or rhythm. According to this perspective of division, there is no inherent relevant information in phonemes per se [1]. Rather, affective information in speech is conveyed either through conventional and arbitrary sound-meaning mappings or through the prosodic features of a vocalization.

However, the long history of poetry, as the most ancient record of human literature, as well as recent empirical results suggest a possible connection between phonemes and another layer of affective meaning beyond the conventional links [2,3,4,5,6]. Stylistic devices such as euphony or cacophony are instructive examples indicating how the sound of a word can evoke a feeling of pleasantness or harshness, respectively. Children already possess the ability to easily evaluate whether a word sounds positive/negative or beautiful/ugly [7]. This idea has been supported by recent experimental evidence highlighting the role of sound in affective meaning making [8], as well as its contribution to the beauty of words [9].

Although the brain networks involved in emotion processing for both verbal and nonverbal stimuli have been well studied, little is known about the neural correlates of the affective potential of a word’s sound (but see [10] for an event-related potential study). In the present study, we examined the neuropsychological reality of sublexical sound effects, and aimed at identifying its underlying brain network. To quantify the affectivity of the sound of words we used a recent psycho-acoustic model [8] which is based on a two-dimensional space of valence (ranging from pleasant to unpleasant) and arousal (ranging from calm to excited) [11,12]. The model relies on the fact that acoustic features characterizing phonemes and their combinations (as in words) are similar to those modulating emotional vocalization and affective prosody (e.g., sound formants, sound intensity). Thus, these specific features extracted from the sound profile of a word can predict affective potential of the sound of that word [8]. Also, previous studies showed a high similarity of acoustic cues to affective judgments across different types of affective sounds (e.g., speech, music, and environmental sound) [13]. Due to this similarity, we hypothesize that affectivity in the sound of a word will be processed in similar brain regions that are involved in processing other types of affective sounds, as proposed by a unifying neural network perspective of affective sound processing [14].

In an fMRI study involving a passive listening task, we presented participants with words varying in their sublexical affectivity (sound) while controlling for lexical (semantic) affectivity. Specifically, we focused on the affective dimension of arousal, as previous studies showed that arousal, compared to valence, can be more reliably decoded and identified from vocal cues [3,8,13,15].

## 2. Materials and Methods

### 2.1. Stimuli

A total of 120 nouns (one to three syllables long) were selected for a 2 × 2 design (30 words for each condition) characterized by an orthogonal twofold manipulation of lexical and sublexical arousal. For lexical arousal we used ratings of words’ affective meaning (min = 1: very low arousing, max = 5 very high arousing) from the normative database BAWL-R [16]. Sublexical arousal was calculated based on features extracted from the acoustic representation of words applying the acoustic model developed in our previous work (see study 2b in [8]). For this, words were uttered in a list-like manner by a professional male actor who was a native speaker of German and recorded with a sampling frequency of 48 kHz and 16 bits per sample. Audio files were then normalized to have the same loudness by matching their root-mean-square (RMS) power. Words were divided into two distinctive conditions of “high” and “low” arousing for each of the factors lexical arousal (‘High’ > 3.25, ‘Low’ <2.75) and sublexical arousal (‘High’ > 3, ‘Low’ < 3), and carefully controlled for relevant psycholinguistic variables across all of four cells of experimental conditions. Lexical arousal (and lexical valence) was closely controlled for between the two cells of sublexical arousal, and vice versa (Table 1). In order to create an acoustic baseline, we randomly selected 16 words from the word material (4 from each condition) and converted them to signal-correlated noise (SCN). Along with our stimulus material (120 words + 16 SCN), a total of 76 additional words (mostly emotionally neutral) were presented which were a part of another study, and were discarded from further analysis here.

### 2.2. Participants

Twenty-nine right-handed German native speakers (17 women, mean age 25.2 years, range: 20–35 years) with no history of neurological or psychiatric illness or any hearing problems volunteered to participate in the study, receiving either 15 Euros or psychology course credit for their participation. Handedness was determined using the Edinburgh Inventory [17].The Ethical Committee of the Freie Universität Berlin had approved the investigation. Informed consent was obtained according to the Declaration of Helsinki.

### 2.3. Procedure

Spoken words were presented via MRI-compatible headphones sufficiently shielded from scanner noise to ensure clear perceptibility. Participants were instructed to pay attention and to carefully listen to the words. A trial began with the presentation of a fixation cross for between 1500 ms and 6500 ms, jittered in steps of 500 ms, in the center of the screen. Jittering durations and the stimulus presentation order over different experimental conditions (HH, HL, LH, LL, SCN, Fillers), were optimized to ensure a maximal signal-to-noise ratio. After presentation of a stimulus the fixation cross disappeared. All blocks were set to a fixed length of 370 volumes. A total number of 10 trial words were presented prior to the experiment, which were excluded from the analysis. Words were split and presented in two runs. Between the two runs the participants could take a break.

### 2.4. fMRI data Acquisition

Imaging data were collected on a Siemens Tim Trio 3T MR scanner. Functional data used a T^*^_2_-weighted echo-planar sequence [slice thickness: 3 mm, no gap, 37 slices, repetition time (TR): 2 s, echo time (TE): 30 ms, flip angle: 70°, matrix: 64 × 64, field of view (FOV): 192 mm, voxel size: 3.0 mm × 3.0 mm × 3.0 mm, 2 × 305 volumes, acquisition time: 2 × 610 s]. At the beginning of the experimental session, magnitude and phase images for the field map were acquired: [slice thickness: 3 mm, no gap, 37 slices, TR: 488 ms, 2 TE: 4.92 and 7.38 ms, flip angle: 60°, matrix: 64 × 64, FOV: 192 mm, voxel size: 3.0 mm × 3.0 mm × 3.0 mm, acquisition time: 65 s]. Individual high-resolution T1-weighted anatomical data (MPRAGE sequence) were also acquired (TR: 1.9, TE: 2.52, FOV: 256, matrix: 256 × 256, sagittal plane, slice thickness: 1 mm, 176 slices, resolution: 1.0 mm × 1.0 mm × 1.0 mm).

### 2.5. Post-Scan Tests

#### 2.5.1. Unannounced Recognition Test

At the end of the experiment, outside the scanner, an unannounced recognition test was performed to assess participants’ involvement in the task and mnemonic effects of the experiment. Participants were presented with the same 120 words used in the scanner (OLD) mixed with 120 new words (NEW), which were matched with OLD items for word frequency, number of letters, number of phonemes, number of syllables, and imageability rating, as well as valence and arousal (selected from the same range as used for OLD items). Participants were asked to rate how confident they were that the presented word was or was not part of the word list in the scanner (from certainly not presented in the scanner = 1 to certainly presented in the scanner = 5).

#### 2.5.2. Ratings

After the recognition test, in two separate rating studies, participants were asked to evaluate the words presented in the scanner for their lexical arousal (study1) and sublexical arousal (study2). For the latter, participants were instructed to only concentrate on the sound aspect of the words while trying to suppress their meaning (cf. [8]).

### 2.6. fMRI Preprocessing

The fMRI data were preprocessed and analyzed using the software package SPM12 (www.fil.ion.ucl.ac.uk/spm). Preprocessing consisted of slice-timing correction, realignment for motion correction, magnetic field inhomogeneity correction through the creation of a field map, and coregistration of the structural image onto the mean functional image. The structural image was segmented into gray matter, white matter, cerebrospinal fluid, bone, soft tissue, and air/background [18]. A group anatomical template was created with DARTEL (Diffeomorphic Anatomical Registration using Exponentiated Lie algebra, [19]) toolbox from the segmented gray and white matter images. Transformation parameters for structural images were then applied to functional images to normalize them to the brain template of the Montreal Neurological Institute (MNI) supplied with SPM. Functional images were resampled to a resolution of 1.5 × 1.5 × 1.5 mm, and spatially smoothed with a kernel of 6 mm full-width-at-half-maximum during normalization.

### 2.7. fMRI Analysis

Voxel-wise fixed effects contrast images made by subtraction analyses were performed at the single subject level and random effects analyses [20] were conducted at the group level to create SPM contrast maps. On the single-subject level, each of the six conditions (HH, HL, LH, LL, SCN, and FILLERS) was convolved with the haemodynamic response function (HRF). Events were modeled as delta functions with zero duration. The beta images of each conditional regressor were then taken to the group level, where a full-factorial second level analysis with the factors lexical arousal and sublexical arousal was used. An unconstrained non-directional 2 × 2 ANOVA whole brain analysis was performed with the factors lexical arousal (High, Low) and sublexical arousal (High, Low), to investigate the overall presence of main and interaction effects. For whole-brain fMRI analyses, we used the cluster defining threshold (CDT) of *p* < 0.005, then applied cluster-level family-wise error (FWE) correction to *p* < 0.05 for the entire image volume, as suggested by Liebermann and Cunningham [21] for studies in cognitive, social and affective neuroscience. The labels reported were taken from the ‘aal’ labels in the WFU Pickatlas Tool. The Brodmann areas (BA) were further checked with the Talairach Client using nearest gray matter search after coordinate transformation with the WFU Pickatlas Tool.

## 3. Results

### 3.1.Behavioral Results

#### 3.1.1. Recognition Test

Across all participants, we performed a Linear Mixed Model analysis predicting the recognition rate, with word category (OLD vs. NEW) as fixed factor and words as well as participants as random factors. Results supported a performance above chance for recognizing OLD words, with a significantly higher score average (M = 3.53) compared to NEW words (M = 2.54): *t* = −20.6, *p* < 0.0001. We next performed simple t-tests to compare the recognition rate between the levels of word category (OLD vs. NEW) separately for each participant. An effect of word category (OLD vs. NEW) on accuracy was observed for 27 participants out of 29 (*t* = 6.4 ± 3.2). These results indicate that the majority of participants had been attentive during the passive listening task. Two participants with a performance not higher than chance level (*t* = 0.28, *t* = 1.14) were consequently excluded from further analyses.

#### 3.1.2. Ratings

**Figure 1:**
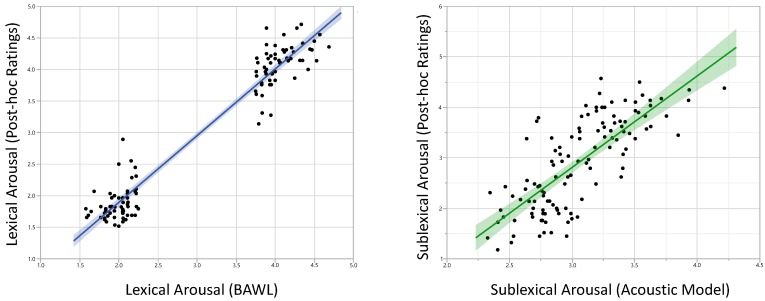
Results of post-scan ratings were highly correlated with affective measures used for the fMRI-experiment. Left: lexical arousal (*r* = 0.97), Right: sublexical arousal (*r* = 0.76).

To check the reliability of our experimental manipulations, we correlated the rating values for lexical and sublexical arousal used for the experiment with our post-scan data. For both, the coefficients were very high: *r* = 0.97, *p* < 0.0001, (*r*_min_ among all participants = 0.73), and *r* = 0.76, *p* < 0.0001 (*r*_min_ among all participants = 0.49), respectively (Figure 1).

### 3.2. Neuroimaging Results

#### 3.2.1. Main Effect of all Words Compared to SCN

The comparison between all words contrasted with the baseline condition of the SCN revealed left-lateralized activations in core language areas, i.e., the inferior frontal gyrus (IFG), middle and superior temporal gyrus, and inferior parietal lobule (BA 40), suggesting that this experiment successfully tapped into the language processing system. Activity was also observed in bilateral parahippocampal gyrus, middle frontal gyrus, and precentral gyrus, as well as the left superior frontal gyrus, the fusiform area, the right caudate, and superior parietal lobule.

#### 3.2.2. Main Effect of the Category Lexical Arousal

**Figure 2:**
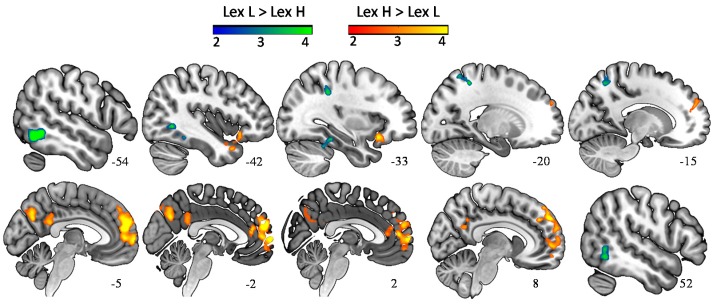
Words with a higher degree of lexical arousal (Lex H > Lex L) elicited stronger activation in a widespread network of medial and inferior frontal gyrus, as well as temporal pole, cuneus, precuneus, and posterior cingulate cortex. The reverse contrast (Lex L > Lex H) resulted in an enhanced BOLD signal in visual and somatosensory cortex (*p* < 0.05, FWE-corr.).

Words with higher levels of lexical arousal (Lex H > Lex L) elicited a large cluster of activation in the left and right dorsolateral and medial prefrontal cortex, a cluster of activation extending from the left IFG into the anterior end of left temporal lobe, as well as a cluster including the left posterior cingulate cortex (PCC) and precuneus (Table 2, Figure 2). Words with lower level of lexical arousal (Lex L > Lex H) elicited a cluster of activation in the left extrastriate cortex in middle occipital gyrus (BA 19) extending to the fusiform area (BA 37) and mirrored by a smaller cluster in the right occipital lobe (BA 37), as well as a cluster of activation immediately posterior to the primary somatosensory cortex (BA 5).

#### 3.3.3. Main Effect of the Category Sublexical Arousal

**Figure 3:**
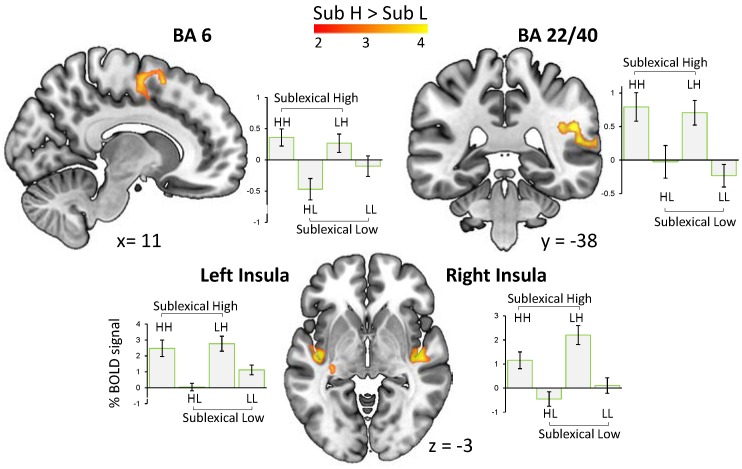
The main effect of sublexical arousal (i.e., words sounding high vs. low arousing) and the related pairwise comparisons were associated with an enhanced BOLD signal in bilateral posterior insula, superior temporal cortex (BA 22 extending to BA40), as well as supplementary and primary motor cortex (BA 6) (*p* < 0.05, FWE-corr.).

Words with higher sublexical arousal (Sub H > Sub L) evoked an increased BOLD signal in bilateral posterior insula, a cluster including the posterior part of superior temporal area and the right supramarginal gyrus, as well as the right premotor cortex and supplementary motor area (Figure 3). No activation was observed for the contrast Sub L > Sub H.

## 4. Discussion

The current study investigated the neural correlates underlying the affective potential of a word’s sound and whether brain regions involved in processing emotional vocalization and affective prosody are also used to process affectivity in the sound of a word.

The overall activation observed for the effect of lexical arousal (Lex H > Lex L) is in accordance with previous findings showing the involvement of dorsolateral and medial prefrontal cortex, as well as PCC, LIFG, and temporal pole in appraisal and general processing of affective stimuli [22,23,24,25]. On the other hand, in the inverse contrast, i.e., Lex L > Lex H, activations of visual and somatosensory areas were observed, suggesting a stronger involvement of perceptual- and image-based systems for processing less emotional words. That is, the semantic processing of words with a lesser emotional connotation is embodied mostly in the brain systems devoted to sensory information about physical word experiences, whereas emotion words are more anchored in affective experiences. This finding is in line with the theories of embodied language stating that concepts are formed as a result of interactions with the real world in various sensory, motor, and affective information about external world experiences (e.g., [26,27,28,29]).

By replicating the results of previous studies for both contrasts, Words > SCN (see Results) and Lex H > Lex L, as well performing an unannounced recognition test, we showed that the present experiment successfully engaged participants in carefully listening to words, thus assuring the reliability of the results, including those of the subsequent effect of sublexical arousal.

Results for the main effect of sublexical arousal (Sub H > Sub L) indicate a substantial sharing between the processing networks for the affectivity in the sound of words and other types of affective sounds. This provides the first neuroimaging evidence for the emotion potential lying in the sound of words, and, importantly, it supports the idea of a unifying neural network of affective sound processing rather than a traditional view that proposes distinct neural systems for specific affective sound types [14]. According to this view, all affective sounds consistently induce brain activity in a common core network which consists of (i) superior temporal cortex and amygdala: likely involved in decoding of affective meaning from sound with amygdala’s involvement rather in less complex stimuli, (ii) frontal and insular regions: likely involved in the evaluation and perception of sound, respectively, and (iii) motor-related areas: likely involved in emotional behavior [14].

The observed activation in the right superior temporal area (BA 22) has been associated, for instance, with intensity of both happy and angry intonations [30]. This effect may be driven by a combination of acoustic features expressing the arousal in the speaker’s voice [31]. Superior temporal areas have been shown to be involved in discriminating sound pitch and sound intensity [32] which are two acoustic features shaping affective prosody [15,33]. Crucially, these two features serve as significant predictors in the acoustic model of sublexical arousal [8] used in the present study. The absence of the activation of amygdala in this part of network may indicate the complexity of speech signals, and is in line with previous findings that show that the amygdala’s involvement in the processing of less complex affective sounds (e.g., non-human environmental sounds, and nonverbal vocalizations), probably due to their function as an emotional signal at a very basic level [14,34]. From the expected response in the fronto-insular brain system, we observed significant clusters of activation in bilateral insula, but no activation in any of the frontal regions.

Concerning the widespread connections of the posterior insula with the auditory cortex and many afferents that it receives from thalamus, previous reports have shown the insula’s significant involvement in auditory temporal processing of most types of emotional sound [14,35,36]. Insula has also been proposed to function as a mediator between sensory and affective brain systems in the perception of affective sounds, thereby enabling a self-experience of emotions in terms of a subjective feeling [14,36]. In regard to the anticipated response in frontal brain regions (e.g., IFG), the absence of such an activation in our study is presumably due to the lack of affective evaluations in the experimental task we used: that is, passive listening. Increasing activation in IFG, as well as its connectivity with STG, is associated with evaluative judgments of affective prosody [37], which our participants were not asked for (but see [38] for a refined fronto-temporal network for the decoding of affective prosody).

In line with the proposed view of a unifying core network, we also observed a cluster of activation in premotor cortex and supplementary motor area. This finding aligns with reports on motor responses to the variety of high arousing sounds [39,40] suggesting that emotionally charged stimuli mobilize the motor system to be prepared to take action for approach or withdrawal. This sound-motion relationship has also been proposed to underlie the feeling of being in the ‘groove’ [41], or a general urge to move when listening to music [35].

## 5. Conclusions

Our study is the first attempt to understand the brain response to the affective potential lying in the sound of words. In accordance with a unifying neural network view for affective sound processing, we observed BOLD responses in superior temporal area, insula, and premotor cortex, suggesting that the affectivity in the sound of words shares a processing network with other types of emotional vocal cues. Our study thus provides the first neuroimaging evidence for a phenomenon that has long been deployed in poetry and the arts, i.e., evoking affective (and aesthetic) responses by the use of certain words with specific sound patterns. Our data also suggests that human subjects are sensitive to the affective information in the sound of words even when the attentional focus is not directed on that aspect.

## Figures and Tables

**Table 1:** Characteristics of word stimuli.

Variable	Word Category								Inferential Statistics
	HH		HL		LH		LL		
	M	SD	M	SD	M	SD	M	SD	
Lexical Arousal	4.07	0.24	4.04	0.22	1.99	0.16	1.99	0.18	F(3,116) = 983, *p* < 0.0001
Lexical Valence	−1.83	0.52	−1.83	0.51	0.22	0.36	0.18	0.37	F(3,116) = 205, *p* < 0.0001
Sublexical Arousal	3.36	0.31	2.76	0.19	3.30	0.27	2.77	0.21	F(3,116) = 50.5, *p* < 0.0001
Word Frequency	0.64	0.75	0.74	0.76	0.57	0.78	0.51	0.75	F(3,116) = 0.47, *p* = 0.69
Imageability	4.78	1.01	4.56	1.0	4.93	0.90	5.02	1.16	F(3,116) = 1.11, *p* = 0.34
Number of Syllables	1.86	0.73	2.1	0.54	2.0	0.69	2.03	0.61	F(3,116) = 0.68, *p* = 0.56
Number of Phonemes	5.3	1.36	5.23	1.10	5.13	0.89	4.93	1.20	F(3,116) = 0.57, *p* = 0.63
duration (ms)	873	116	850	102	826	108	836	100	F(3,116) = 1.06, *p* = 0.36

HH = High-High, HL = High-Low, LH = Low-High, LL = Low-Low: the first letter indicates the lexical and the second sublexical arousal.

**Table 2:** Results for two main contrasts of *lexical* and *sublexical* arousal.

Contrast	Anatomical Definition		MNI Coordinates			Z	K
			x	y	z		
LexH > LexL	L/R	Medial Frontal Gyrus (BA 9)	−3	56	20	5.12	4079
	L	IFG (BA 47), Temporal Pole (BA 38)	−30	21	−17	4.48	672
	L/R	Cuneus, Precuneus (BA 7, BA31)	−3	−68	32	4.01	694
	L	Posterior Cingulate Cortex (BA 23)	−8	−47	26	3.90	492
LexL > LexH	L	Middle Occipital Gyrus (BA 37, 19)	−53	−60	−11	5.88	1244
	R	Middle Occipital Gyrus (BA 37)	56	−57	−8	3.88	515
	L	Somatosensory Cortex (BA 5)	−21	−47	54	4.39	717
SubH > SubL	L	Posterior Insula (BA 13)	−42	−15	−1.5	4.86	861
	R	Posterior Insula (BA 13)	39	−15	1.5	4.78	943
	R	Superior Temporal Area (BA 40, BA 22)	51	−38	24	4.58	852
	R	Supplementary and Premotor area (BA 6)	12	−6	54	3.87	524

Significant peak voxel for all comparisons at *p* < 0.05 FWE-corrected: H = High arousal, L = Low Arousal, Lex = Affective lexical meaning of arousal, Sub = Affective sublexical sound of arousal. MNI = Montreal Neurological Institute, IFG = inferior frontal gyrus, L/R = Left/Right.
